# Collagenous Alzheimer amyloid plaque component impacts on the compaction of amyloid-β plaques

**DOI:** 10.1186/s40478-020-01075-5

**Published:** 2020-12-07

**Authors:** Tadafumi Hashimoto, Daisuke Fujii, Yasushi Naka, Mayu Kashiwagi-Hakozaki, Yuko Matsuo, Yusuke Matsuura, Tomoko Wakabayashi, Takeshi Iwatsubo

**Affiliations:** 1 Department of Neuropathology, Graduate School of Medicine, The University of Tokyo, 7-3-1 Hongo, Bunkyo-ku, Tokyo, 113-0033 Japan; 2 Department of Innovative Dementia Prevention, Graduate School of Medicine, The University of Tokyo, 7-3-1 Hongo, Bunkyo-ku, Tokyo, 113-0033 Japan; 3 Department of Neuropathology and Neuroscience, Graduate School of Pharmaceutical Sciences, The University of Tokyo, 7-3-1 Hongo, Bunkyo-ku, Tokyo, 113-0033 Japan

**Keywords:** Alzheimer’s disease/amyloid, β peptide/CLAC/senile plaques

## Abstract

Massive deposition of amyloid β peptides (Aβ) as senile plaques (SP) characterizes the brain pathology of Alzheimer’s disease (AD). SPs exhibit a variety of morphologies, although little is known about the SP components that determine their morphology. Collagenous Alzheimer amyloid plaque component (CLAC) is one of the major non-Aβ proteinaceous components of SP amyloid in AD brains. Here we show that overexpression of CLAC precursor (CLAC-P) in the brains of APP transgenic mice results in a significant remodeling of amyloid pathology, i.e., reduction in diffuse-type amyloid plaques and an increase in compact plaques laden with thioflavin S-positive amyloid cores. In vivo microdialysis revealed a significant decrease in Aβ in the brain interstitial fluid of CLAC-P/APP double transgenic mice compared with APP transgenic mice. These findings implicate CLAC in the compaction of Aβ in amyloid plaques and the brain dynamics of Aβ.

**Electronic supplementary material:**

The online version of this article (10.1186/s40478-020-01075-5) contains supplementary material, which is available to authorized users.

## Introduction

Senile plaques (SPs) are the pathological hallmark lesions in the brains of patients with Alzheimer’s disease (AD), of which the major building blocks are amyloid fibrils composed of amyloid-β peptides (Aβ) [17]. Aβ is a proteolytic product of the sequential cleavage of Aβ precursor protein (APP) by β- and γ-secretases. Genetic evidence [16, 34], together with cellular and animal studies [6, 15, 23, 48], led to the amyloid hypothesis, which assumes that fibrillization and deposition of Aβ is central to the pathogenesis of AD [41]. However, factors that trigger the process of Aβ deposition remain yet to be characterized.

Morphologically, SPs are classified into several types [11, 12, 26, 57]: “Primitive” plaques are distinctly circumscribed structures without amyloid cores; “Typical” or “classical” plaques harbor amyloid cores at the center, and “burnt-out” plaques composed exclusively of the amyloid cores. Amyloid cores are stained by the dyes specifically bound to β-sheeted structures, e.g., thioflavin S (ThS). Swollen dystrophic neurites are associated with the primitive and typical plaques, which are interpreted as signatures of local amyloid toxicity [31, 42]. Some reports have suggested the correlation between the ThS-positive mature plaques and neurotoxicity and synaptic dysfunction in the brains of AD patients and APP transgenic (tg) mice, implicating the role of mature plaques in the pathological progression of AD [43, 52]. In contrast to the “mature” plaques, “diffuse” plaques composed of amorphous Aβ deposits that are negative for ThS and lack the association of dystrophic neurites [58]. Because diffuse plaques are observed at the earliest stage of Aβ deposition during the progression of AD pathology [24, 25], they are considered as the most “immature” form of Aβ deposits. However, specific components of amyloid deposits that determine the SP morphology remain unidentified.

We have previously identified CLAC (collagenous Alzheimer amyloid plaque component) in SPs of AD brains [18]. CLAC is a proteolytic fragment cleaved by furin convertase from a membrane-spanning collagen CLAC-P (CLAC precursor)/collagen type XXV α1 chain (COL25A1), the latter being shown to play a crucial role in intramuscular motor innervation during neuromuscular development [35, 50]. Genetically, SNPs in *COL25A1* gene have been shown to be associated with AD in a Swedish cohort [14], and rare coding variants in *COL25A1* gene were identified in the longevity population of the Wellderly healthy aging cohort [13]. These genetics findings also led us to hypothesize that CLAC is involved in the pathogenesis of AD.

CLAC exhibits a unique pattern of distribution in AD brains: primitive SPs and the peripheral portion of typical SPs are strongly positive for CLAC, whereas diffuse plaques, cerebral amyloid angiopathies and the core portion of typical SPs are CLAC-negative [27]. A number of proteomics analyses also revealed the existence of CLAC in the isolated SP fractions [28, 29, 45], formic-acid-extracts [55], or sarkosyl-insoluble-extracts [2] of AD brains. These results support the view that CLAC may be involved in the morphological variation of SPs in AD brains.

In this study, we attempted to verify this hypothesis in tg mice overexpressing human CLAC-P and human APP. In the brains of APP/CLAC-P double tg mice, diffuse Aβ plaques were replaced by compact Aβ plaques, and in vivo microdialysis revealed a significant decrease in the levels of Aβ in the brain interstitial fluid. These results support the notion that co-deposition of CLAC with Aβ elicits the remodeling of amyloid deposition into compact plaques, thereby altering the dynamics of Aβ in the brain.

## Materials and methods

### Experimental design

Heterozygous CLAC-P tg mice and APP tg mice were crossed with C57BL/6J wild-type mice. All mice analyzed in this paper were F1 progeny derived from a single cross between heterozygous CLAC-P tg mice and heterozygous APP tg mice. The whole brains ware removed from the skull, bisected along the sagittal plane, and the right hemisphere was immediately frozen in liquid nitrogen for biochemical analyses and the left hemisphere was immediately fixed in 50 mM phosphate buffer containing 4% paraformaldehyde pH 7.4 for immunohistochemical analyses. For the analyses of J20 × CLAC mice, we analyzed 9 mice (3 female, 6 male) for 3-month-old, 31 mice (21 female, 10 male) for 6-month-old, 18 mice (6 female, 12 male) for 9-month-old, 40 mice (15 female, 25 male) for 12-month-old, and 24 mice (9 female, 15 male) for 15-month-old. For the analyses of A7 × CLAC mice, we analyzed 48 mice (26 female, 22 male) for 18–22-month-old, and 8 mice (8 male) for 5–7-month-old. Detailed genotype of those mice was described in the corresponding figure legend.

### Animals

Human full-length CLAC-P cDNA [18] was subcloned into the *XhoI* site of exon 3 of the Thy1.2 expression cassette. Linearized cDNA by *NotI* digestion was purified and microinjected into pronuclei of single-cell embryos derived from hybrid mice between C57BL/6N male × BDF1 female (Oriental Yeast). CLAC-P tg mice were identified and genotyped by genomic PCR analyses of tail DNA using specific primers: thyfw2 5′-aggtattcatcatgtgctcc-3′ and clnc1rev 5′-atcaggcagcagatgaatgg-3′ (407 base-pairs DNA is amplified). APP tg mice (J20 line) were purchased from Jackson Laboratory and genotyped according to the breeder’s protocol. APP tg mice (A7 line) were generated previously [56] and genotyped using specific primers: TgF1 5′-ctgaggtattcatcatgtgc-3′ and TgR3 5′-ggacattcatgtgcatgttc-3′ (336 base-pairs DNA is amplified). All mice were kept under SPF conditions and fed a regular diet (Oriental Yeast).

### RT-PCR

Mouse tissues were extracted by Isogen (NIPPON GENE) and reverse-transcribed as described [18]. RT-PCR analysis was performed by SuperScript II One-step RT-PCR with PLATINUM Taq (Thermo Fisher Scientific) using human CLAC-P specific primers: forward 5′-tccattcatctgctgcctgataccc-3′ and reverse 5′-tcaggcggcgtttaatgagctgctg-3′ (321 base-pairs DNA is amplified).

### Antibodies

Polyclonal antibodies against human CLAC-P were previously described [18]. A monoclonal antibody 82E1 is a human Aβ N-terminal-end specific antibody (Immuno-Biological Laboratories). A monoclonal antibody BAN50 against human APP and Aβ were described [1, 48]. A monoclonal antibody 6E10 against human APP and Aβ was purchased from BioLegend. A monoclonal antibody against ubiquitin was purchased from DAKO. A polyclonal antibody against RFP was purchased from MBL. A polyclonal antibody against Iba1 was purchased from Wako. Primary antibodies were applied at dilutions of 1:200 for anti-NC1; 1:100 for anti-NC2-2; 1:1000 for anti-NC3 and anti-NC4; 1:500 for anti-pyroE113; 1:100 for 82E1; 1:5000 for BAN50; 1:1000 for 6E10; 1:500 for anti-ubiquitin; 1:1000 for anti-RFP; 1:500 for anti-Iba1.

### Immunoblot analysis and two-site enzyme-linked immunosorbent assay (ELISA)

Hemispheres of mouse brains were homogenized in 10 volumes (w/v) of TBSI buffer (50 mM Tris–HCl pH 7.6, 150 mM NaCl, 0.5 mM diisopropyl fluorophosphates, 0.5 mM phenylmethylsulfonyl fluoride, 1 mM EGTA, 1 μg/ml antipain, 1 μg/ml leupeptin, 1 μg/ml pepstatin, 1 μg/ml Nα-Tosyl-_l_-lysine chloromethyl ketone, or 50 mM Tris–HCl pH 7.6, 150 mM NaCl, with a cOmplete protease inhibitors cocktail (Roche)), and centrifuged at 260,000 × g for 20 min at 4 °C. The supernatant was used as a TBS-soluble fraction or a cytosolic fraction. The pellet was homogenized again in 10 volumes (w/v) of TBSI buffer containing 2% Triton X-100 and centrifuged at 260,000× *g* for 20 min at 4 °C. The supernatant was used as a membrane fraction. The pellet was homogenized again in 10 volumes (w/v) of TBSI buffer containing 2% SDS, incubated at 37 °C for 30 min and centrifuged at 260,000× *g* for 20 min at 20 °C. The SDS-insoluble pellet was dissolved in 1 ml of 70% formic acid, centrifuged at 260,000× *g* for 20 min at 4 °C. The formic acid-soluble fraction was desiccated by Speed-Vac and then resuspended 1 volume (w/v) of dimethyl sulfoxide (DMSO). The DMSO-soluble fraction was used as a SDS-insoluble fraction. SDS-PAGE was performed as previously described [18], under a reducing condition. The immunoblots were developed using Immunostar reagents (Wako) or SuperSignal west femto (Thermo Fisher Scientific) and visualized by LAS-1000plus (FUJIFILM). The intensity of the band was quantified by Image Gauge software (FUJIFILM). The amount of TBS-soluble or SDS-insoluble human Aβ was quantified using a Human/Rat Aβ40 or Aβ42 specific two-site ELISA (BNT77/BA27 for Aβ40 or BNT77/BC05 for Aβ42, FUJIFILM Wako) as previously described [51].

### Immunohistochemistry and ThS staining

Mice hemispheres were immediately fixed in 50 mM phosphate buffer containing 4% paraformaldehyde pH 7.4 for 24 h. The paraffin-embedded sections cut at 5 μm were de-paraffinized in xylene and rehydrated through an ethanol dilution series. For immunostaining of Aβ, the sections were pretreated microwave (550 W) in citrate buffer pH 6.0 for 10 min, following reaction with 100 μg/ml of Proteinase K (Worthington) in Tris-buffered saline (TBS) pH 7.6 for 7 min. For immunostaining of CLAC, the sections were pretreated microwave as above. Primary antibodies were treated for 12 h. HRP-conjugated anti-mouse or anti-rabbit antibody was used as a secondary antibody (Vector Laboratories). The paraffin sections were then visualized with VECTASTAIN ABC elite system (Vector Laboratories) using diaminobenzidine [27]. Images were captured by HC-2500 digital image recording system (FUJIFILM) mounted on a BX51 microscope (Olympus). The mean area of amyloid burden in hippocampus stained by BAN50 or 82E1 from 3 images per an animal was measured using MacSCOPE software (Mitani). The mean number of ubiquitin-positive plaques in hippocampus from 3 images per an animal was also quantified using MacSCOPE software. The mean plaque size or circularity of each Aβ plaque in the hippocampus was quantified using Image J software. For ThS staining, the paraffin sections were de-paraffinized, rehydrated and stained in 1% ThS (Sigma) containing distilled water for 10 min. In double-labeling experiments for CLAC and ThS, paraffin sections were fluorescent-labeled by anti-NC2-2, followed by ThS staining. ThS images were observed with Olympus fluoview confocal microscope as described [27] and captured by DP70 digital image recording system (Olympus). The mean number of ThS-positive plaques in hippocampus from 3 images per an animal was quantified using MacSCOPE software.

### Adeno-associated virus serotype 9 (AAV9)-mediated expression of CLAC-P

pAAV.hSyn.EGFP.WPRE.bGH vector is a gift from James M. Wilson (Addgene plasmid #105539; http://n2t.net/addgene:105539; PRID:Addgene_105539). The cDNA encoding nuclear localization signal-tagged dTomato-P2A-3xFLAG-tagged CLAC-P fusion protein was substituted into EGFP sequence in the pAAV.hSyn.EGFP.WPRE.bGH vector that carries human Synapsin I promoter. The packing, purification, or titer determination of AAV9-CLAC-P was performed by the PENN Vector Core. The stereotaxic injection of AAV9-CLAC-P into cortex and hippocampus of APP tg mice was performed as described [22]. Briefly, 1 μl of AAV9-CLAC-P (5.0 × 10^12^ genome copy/ml) solution or phosphate-buffered saline (PBS) was injected into the hippocampus (anterior–posterior − 2.5 mm, medial–lateral ± 2.0 mm, dorsal–ventral − 1.8 mm from Bregma) and the cortex (anterior–posterior − 2.5 mm, medial–lateral ± 2.0 mm, dorsal–ventral − 1.0 mm from Bregma) in 14-month-old APP tg mice at a rate of 0.1 μL/min. After four months from injection, AAV-injected mice were analyzed by immunohistochemistry or ThS staining.

### In vivo microdialysis

In vivo microdialysis was performed as previously [59]. Briefly, a microdialysis cannula (Eicom) was inserted stereotaxically into the hippocampal area of APP tg mice (A7 line) or APP/CLAC-P double tg mice filled with artificial cerebrospinal fluid (aCSF; 1.3 mM CaCl_2_. 1.2 mM MgSO_4_, 3.0 mM KCl. 0.4 mM KH_2_PO_4_, 25 mM NaHCO_3_, 122 mM NaCl) containing 0.15% bovine serum albumin through a cranial hole 2.8 mm posterior and 0.5 mm lateral to the right side from the bregma, with an angle of 37.5º to the depth of 1.3 mm, and set with a dialysis probe of molecular-weight cut-off at 1,000 kDa (Eicom). aCSF was circulated at the rate of 1.3 μl/min and sampling was started after 3 h, with mice in a free moving cage. Levels of Aβ42 in ISF were quantified by a Human/Rat Aβ42 specific two-site ELISA (BNT77/BC05, FUJIFILM Wako).

### Statistical analyses

Tests for statistical significance between groups were performed using StatView software (SAS) or Prism 6 (GraphPad). In experiments involving two groups, Mann–Whitney U test for non-parametric tests, and Student’s *t* test, paired t-test, or Welch’s t-test for parametric tests were performed. The details and results of all statistical tests are described in the corresponding figure legend for each experiment.

## Results

### Overexpression of CLAC-P did not alter the expression and processing of APP in the brains of APP/CLAC-P double tg mice

**Fig. 1:**
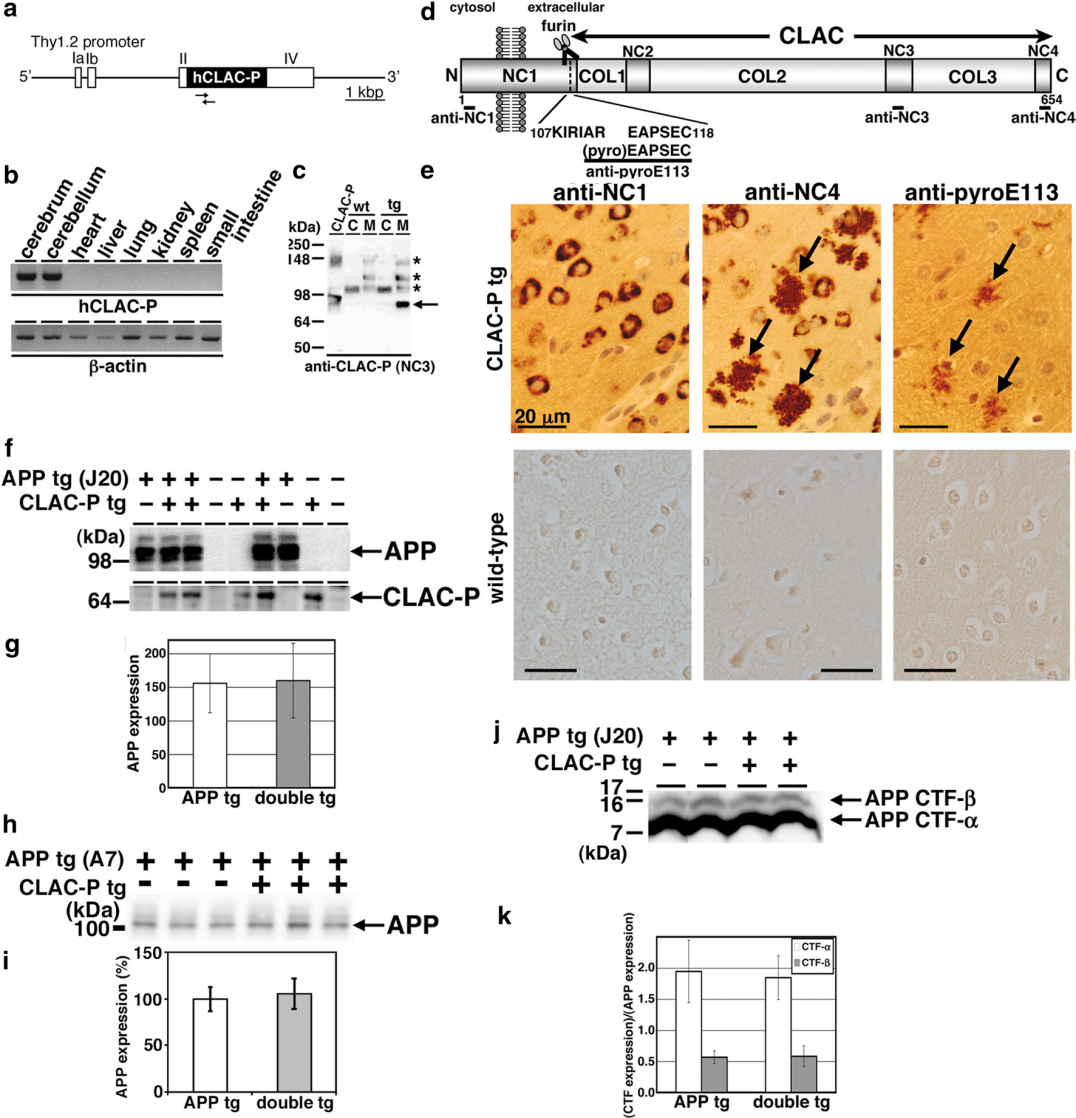
Generation of CLAC-P tg mice and APP/CLAC-P double tg mice. **a** A schematic structure of human CLAC-P (hCLAC-P) transgene using murine Thy1.2 promoter. The arrows show the location of human CLAC-P specific primers. Scale bar shows 1 kbp. **b** RT-PCR analyses in CLAC-P tg mice (line#74) using human CLAC-P (upper panel) and murine β-actin (lower panel) specific primers. **c** Immunoblot analysis of the brains of CLAC-P tg and its littermate wild-type mice (wt). Mice brains were extracted sequentially by Tris saline (cytosolic fraction, C) and 2% Triton X-100 (membrane fraction, M). Arrow shows the mobility of CLAC-P in HEK293 cells. Asterisks show the non-specific bands. **d** Schematic representation of the domain structure of human CLAC-P. The NC1 domain composed of cytoplasmic, transmembrane and extracellular portions, three extracellular non-collagenous domains (NC2, NC3 and NC4) and the three collagenous domains (COL1, COL2 and COL3) are shown. The epitope locations of the antibodies are shown in black bars. **e** Immunohistochemical analyses of cerebral cortex of 6-month-old CLAC-P tg (upper panels) or wild-type mice (lower panels) by anti-NC1, NC4, and pyroE113 human CLAC-P specific antibodies. The plaque-like structures are exclusively positive for the extracellular epitope of CLAC-P (arrows). Scale bar shows 20 μm. **f**, **h** Immunoblots of human APP and human CLAC-P in membrane fraction of the brains of double (J20 (**f**), A7 (**h**)) tg mice by anti-human APP antibody (BAN50 upper panel in **f**, 6E10 in **h**), or anti-CLAC-P antibody (anti-NC4 lower panel in (**h**)). **g** Densitometric analyses of the levels of full-length APP in the brains of APP (J20) and double tg mice. The mean ± SD N = 6 (APP), 7 (double). Student’s t-test, *p* = 0.89. **i** Densitometric analyses of the levels of full-length APP in the brains of APP (A7) and double tg mice. The mean ± SD is shown. N = 13 (APP), 9 (double). Student’s t-test, *p* = 0.90. **j** Immunoblot analysis of the APP CTFs (APP CTF-α and APP CTF-β) in the brains of APP (J20) and double tg mice by anti-APP C-terminus. **k** Densitometric analyses of the levels of APP CTFs (normalized by the levels of full-length APP). The mean ± SD N = 4 (APP), 3 (double) tg. Student’s t-test, *p* = 0.58 (APP CTF-α) and 0.71 (APP CTF-β)

To elucidate the pathological roles of CLAC in vivo, we generated tg mice overexpressing human CLAC-P (CLAC-P tg) in a neuron-specific manner under murine Thy1.2 promoter (Fig. 1a) [47, 54]. We examined the expression levels of human CLAC-P protein in the brains of F2 generation derived from eight F0 founders by immunoblotting and immunohistochemistry, and obtained two high-expressor lines, of which we used the line #74 throughout the study. Human *CLAC*-*P* mRNA was expressed in the central nervous system of CLAC-P tg mice (Fig. 1b), and human CLAC-P protein was recovered from the membrane fraction of the brain (Fig. 1c). We previously showed that CLAC-P is cleaved by furin convertase after the domain _107_KIRIAR_112_ to release its extracellular domain as a secreted form of CLAC-P (sCLAC), which co-deposits with Aβ in AD brains as CLAC [18]. We also found that the amino terminus of CLAC deposited in AD brains underwent pyroglutamate modification [18]. To confirm whether CLAC is secreted in the brains of CLAC-P tg mice, we immunostained slices of brains from CLAC-P tg or wild-type mice using multiple anti-CLAC-P antibodies that specifically recognize the intracellular domain (anti-NC1), extracellular domain (anti-NC4), or the pyroglutamate modified E_113_ (anti-pyroE113) (Fig. 1d). We found that overproduced CLAC-P proteins were localized in neuronal cell bodies throughout the central nervous system of CLAC-P tg mice (Fig. 1e). Notably, tiny plaque-like structures that are positively labeled by anti-NC4 as well as by anti-pyroE113 antibodies, but not by anti-NC1 antibody, were found in the neocortices of CLAC-P tg mice over the age of 3-month-old (Fig. 1e). Such plaque-like structures were not observed by immunostaining with anti-NC4 and anti-pyroE113 antibodies in the brains of wild-type mice (Fig. 1e). These observations suggest that CLAC is secreted from neurons and forms aggregates in the extracellular space of brain parenchyma of CLAC-P tg mice.

To elucidate the effects of CLAC on AD-related pathology in vivo, we crossed CLAC-P tg mice with APP tg mice, which express human APP in neurons and exhibit AD-like amyloid pathology in the brain. For this purpose, we used two lines of APP tg mice, J20 and A7. J20 expresses human APP carrying the Swedish and Indiana mutations (KM670/671NL + V717F) driven under the control of platelet-derived growth factor β-chain promoter [33], and A7 expresses human APP carrying the Swedish and Austrian mutations (KM670/671NL + T714I) under the control of Thy1.2 promoter [56]. F1 progeny derived from a single cross between heterozygous CLAC-P tg mice and heterozygous APP tg mice was used throughout the study.

Immunoblotting of the brain lysates showed comparable levels of human APP between APP single tg mice (J20 or A7 line) and APP/CLAC-P double tg (double tg) mouse littermates (Fig. 1f (J20 line), 1g, 1h (A7 line), 1i). APP undergoes proteolytic cleavage by ectodomain sheddases, i.e., α-secretase or β-secretase, followed by intramembrane cleavage by γ-secretase. We found that the amounts of the carboxy-terminal fragments (CTFs) of APP by α-secretase (CTF-α) or β-secretase (CTF-β) cleavage in the membrane fractions of mouse brains were at similar levels (Fig. 1j (J20 line), 1 k). These data suggest that co-expression of additional single-span membrane protein, CLAC-P, does not affect the expression and processing of APP in the brains of the double tg mice.

### Overexpression of CLAC-P altered the morphology of Aβ plaques in the brains of double tg mice

**Fig. 2:**
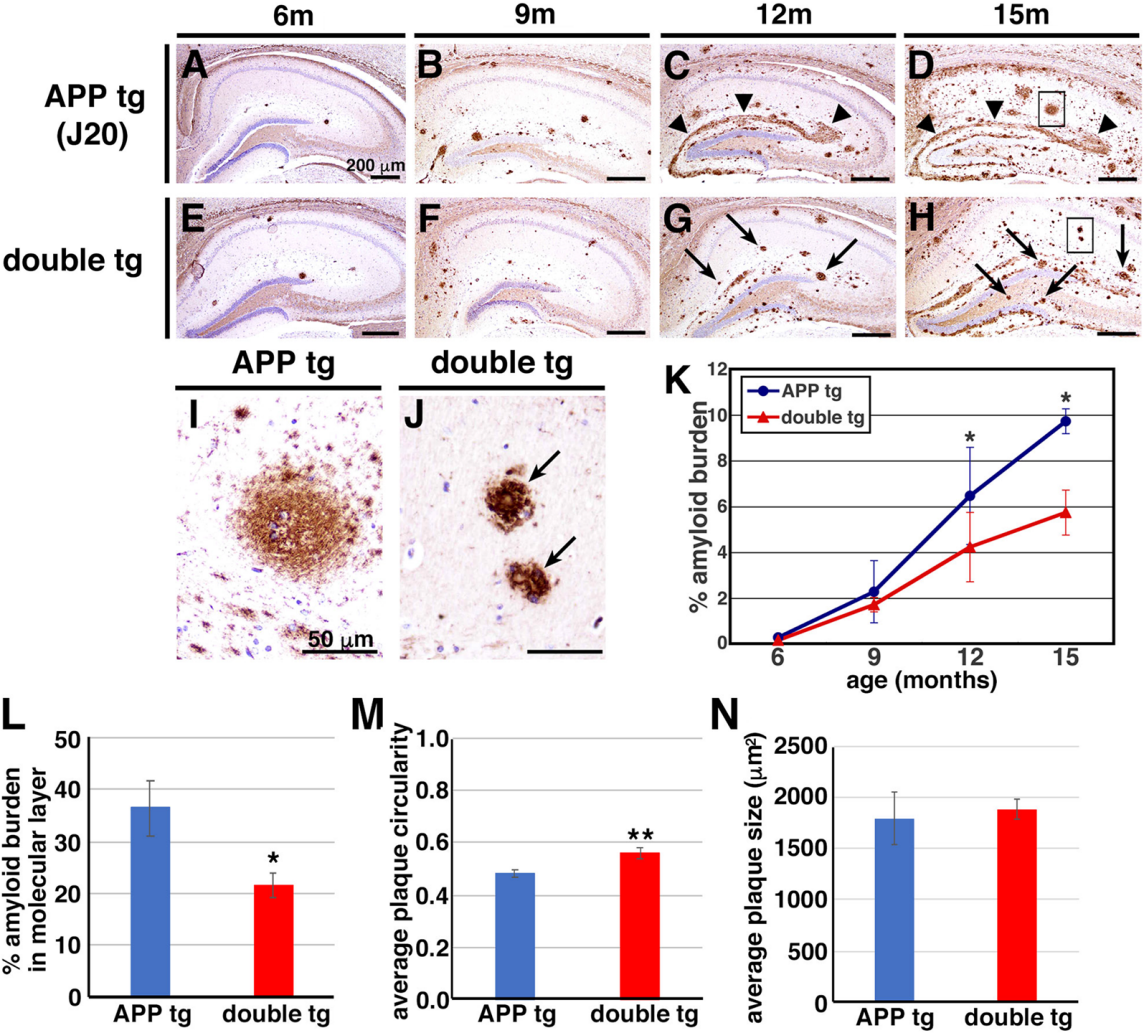
Overexpression of CLAC-P altered the morphology of Aβ deposits (J20 line). **a**–**h** Immunohistochemical analyses of the brains of 6- (**a** and **e**), 9- (**b** and **f**), 12- (**c** and **g**) and 15- (**d** and **h**) month-old APP tg mice (J20 line, **a**–**d**) and double tg mice (**e**–**h**) using an anti-human Aβ antibody (82E1). High magnification images (insets in **d** and **h**) were shown in **i** and **j**, respectively. Diffuse amyloid plaques (**c** and **d**, arrowheads) and huge plaques (**i**) were shown in the hippocampi of APP tg mice. On the other hand, numerous middle-sized plaques were predominant in the hippocampi of 12- and 15-month-old double tg mice (**g**, **h** and **j**, arrows). Scale bar shows 200 μm (**a**–**h**) and 50 μm (**i** and **j**). **k** Quantitative analysis of the amyloid burden (%Aβ immunoreactive areas) in the hippocampus of APP and double tg mice. N = 4, 4, 13, 7 for 6-, 9-, 12-, 15-month-old APP tg, respectively. N = 6, 3, 11, 7 for 6-, 9-, 12-, 15-month-old double tg, respectively. Mann–Whitney U test, *p* = 0.29 (6-month-old), 0.48 (9-month-old), 0.019 (12-month-old), 0.018 (15-month-old). **l** Quantitative analysis of the amyloid burden at the molecular layer of hippocampal dentate gyrus of 15-month-old APP and double tg mice. N = 6 (APP tg) and 7 (double tg). Student’s t-test, *p* = 0.024, *, *p* < 0.05 (**m**, **n**) Average circularity (**m**) and size (**n**) of Aβ plaques in the hippocampus of 15-month-old APP and double tg mice. Note that Circularity value of 1.0 indicates a perfect circle. N = 6 (APP tg) and 7 (double tg). Student’s t-test, *p* = 0.0076, **, *p* < 0.01 (**n**) N = 6 (APP tg) and 7 (double tg). Student’s t-test, *p* = 0.76

We next examined the histopathology of brains of the APP (J20 line) and double tg mice with special attention to the morphology and distribution of Aβ plaques. We immunostained the tissue sections from brains of 6, 9, 12 and 15-month-old APP tg and their littermate double tg mice by an anti-Aβ antibody 82E1. At 6 months of age, a few Aβ plaques appeared at the dentate gyrus, CA1 and CA3 regions in the hippocampus of APP and double tg mice in an almost similar pattern (Fig. 2a, e), consistent with the previous description that Aβ plaques are initially detected in the hippocampus of J20 line at 5–7 months of age [33], suggesting that overexpression of CLAC-P does not influence the onset age of Aβ deposition. After 12 months of age, there emerged a clear difference in the pattern of amyloid deposition between APP and double tg mice: in the hippocampus of APP tg mice, diffuse-type Aβ plaques were observed in a laminar pattern in the molecular layer of the dentate gyrus, whereas diffuse Aβ plaques were rarely observed in the hippocampal area of double tg mice (Fig. 2c, d, g, h). In addition, a few round and well-circumscribed, huge Aβ plaques (> 50–100 μm in diameter) were observed in the hippocampus of APP tg mice (Fig. 2i). In sharp contrast, middle-sized Aβ plaques (< 50 μm in diameter) reminiscent of primitive-type plaques in AD brains, or middle-sized Aβ plaques occasionally laden with amyloid cores resembling typical mature plaques in AD brains, were predominantly observed in the hippocampal region of double tg mice (Fig. 2j). Quantification of the amyloid burden (percent area covered by Aβ immunoreactivity) of the hippocampus showed that the burden was significantly smaller in the double tg mice by ~ 35% at 12 M (mean values: 6.46% in APP and 4.22% in double tg mice) and by ~ 41% at 15 M (mean values: 9.72% in APP and 5.73% in double tg mice) compared with those in APP tg mice (Fig. 2k). Especially, the amyloid burden at the molecular layer of hippocampal dentate gyrus, where diffuse-type plaques were predominant, was significantly smaller in the double tg mice by ~ 41% at 15 M (mean values: 36.4% in APP and 21.7% in double tg mice) (Fig. 2l). It has been documented that compact plaques yield a higher circularity compared with diffuse-type plaques in the brains of APP tg mice (circularity value of 1.0 indicates a perfect circle) [60]. Further detailed observation of the morphology of individual plaques revealed that the mean circularity in plaques in the brains of double tg mice was significantly higher than that of APP tg mice (mean values: 0.48 in APP and 0.56 in double tg mice) (Fig. 2m), without significant differences in the mean plaque size (mean values: 1799 μm^2^ in APP and 1889 μm^2^ in double tg mice) (Fig. 2n). These results suggest that overexpression of CLAC-P decreased the total area (i.e., amyloid burden) of Aβ deposits, and altered the plaque morphology from diffuse-type to well-circumscribed, compact plaques.

**Fig. 3:**
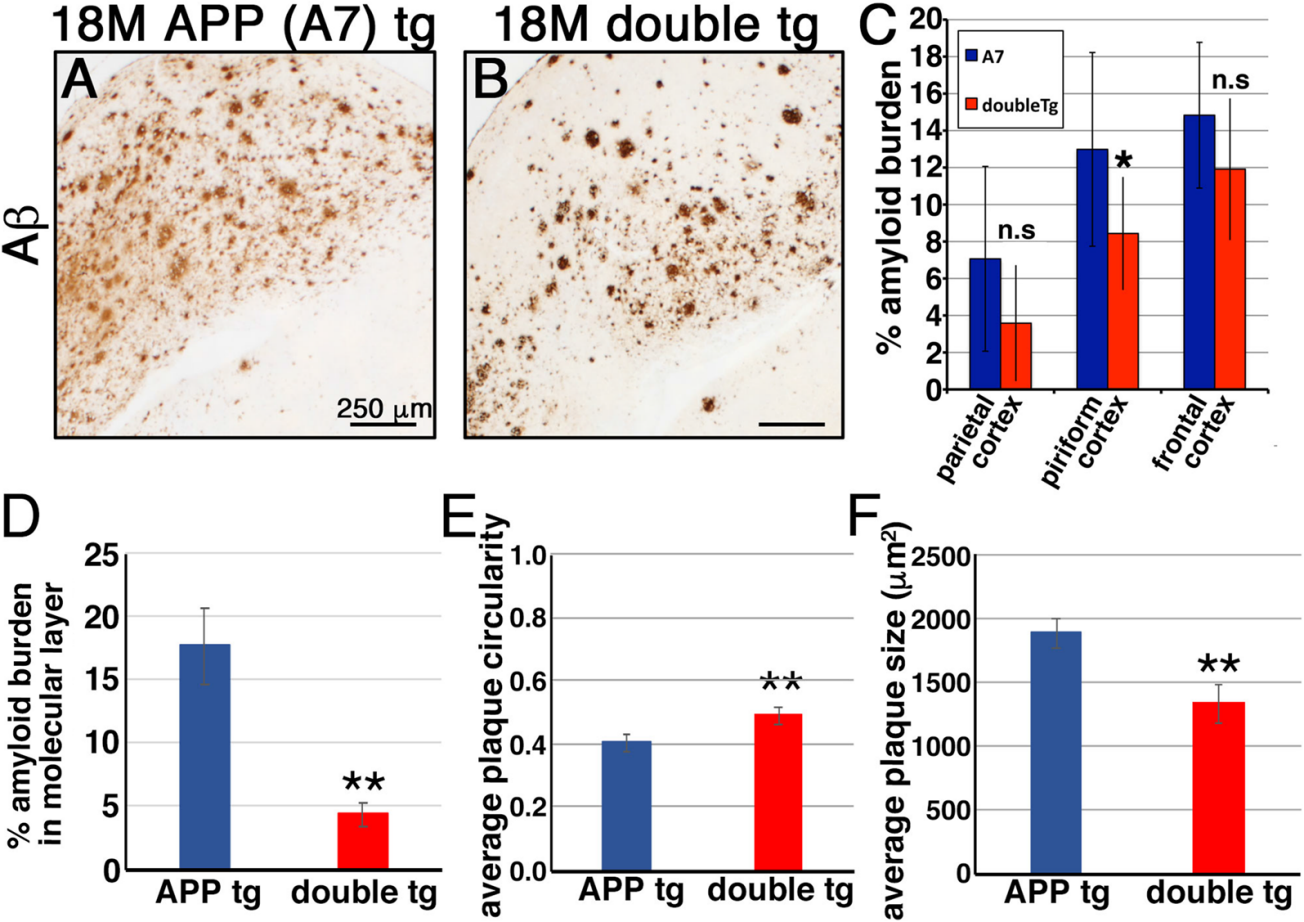
Overexpression of CLAC-P altered the morphology of Aβ plaques (A7 line). **a**, **b** Immunohistochemical analyses of the entorhinal cortex of 18-month-old APP tg (A7, A) and double tg (A7 × CLAC-P, B) mice using an anti-human Aβ antibody (82E1). Scale bar shows 250 μm. **c** Quantitative analysis of the amyloid burden (% Aβ immunoreactive areas) in the parietal cortex, piriform cortex and frontal cortex of APP tg and double tg mice. N = 4, Student’s t-test, *p* = 0.069 (parietal cortex), 0.024 (piriform cortex), 0.10 (frontal cortex), * *p* < 0.05. **d** Qantitative analysis of the amyloid burden at the molecular layer of hippocampal dentate gyrus of 18-month-old APP and double tg mice. **e** N = 15 (APP tg) and 12 (double tg). Student’s t-test, *p* = 0.00061, **, *p* < 0.01. **e**, **f** Average circularity (**e**) and size (**f**) of Aβ plaques in the hippocampus of 18-month-old APP and double tg mice. N = 15 (APP tg) and 12 (double tg). Student’s t-test, *p* = 0.0061, **, *p* < 0.01 (**f**) N = 15 (APP tg) and 12 (double tg). Student’s t-test, *p* = 0.0046, **, *p* < 0.01

We further generated an additional line of double tg mice by crossing CLAC-P tg mice with another type of APP tg mice (A7 line). In the brains of 18-month-old APP tg mice and their littermate double tg mice, Aβ plaques were observed both in the cortex area of APP and double tg mice (Fig. 3a, b). Similarly to J20-based double tg line, middle-sized, well-circumscribed Aβ plaques that were occasionally associated with amyloid cores were predominant and the diffuse-type plaques were hardly observed in the cortex of double tg mice. The amyloid burden in the piriform cortex of double tg mice was significantly smaller compared with those in APP tg mice (Fig. 3c). Furthermore, the amyloid burden at the molecular layer of hippocampal dentate gyrus was significantly smaller in the double tg mice by ~ 76% (mean values: 17.6% in APP and 4.30% in double tg mice) (Fig. 3d). The mean circularity in plaques in the brains of double tg mice was significantly higher than that of APP tg mice (mean values: 0.40 in APP and 0.49 in double tg mice) (Fig. 3e), and the mean plaque size in double tg mice was significantly smaller than that in APP tg mice (mean values: 1891 μm^2^ in APP and 1336 μm^2^ in double tg mice) (Fig. 3f). Thus, similar changes in the total Aβ-positive area and plaque morphology were observed in the double tg mice based on distinct types of APP tg mice, i.e., a substantial decrease in the diffuse-type Aβ plaques and the appearance of middle-sized compact Aβ plaques occasionally with amyloid cores. This strongly suggested that the overexpression of CLAC-P altered the morphology of Aβ plaques into a more compact form.

### Co-deposition of CLAC with Aβ plaques in the brains of double tg mice

**Fig. 4:**
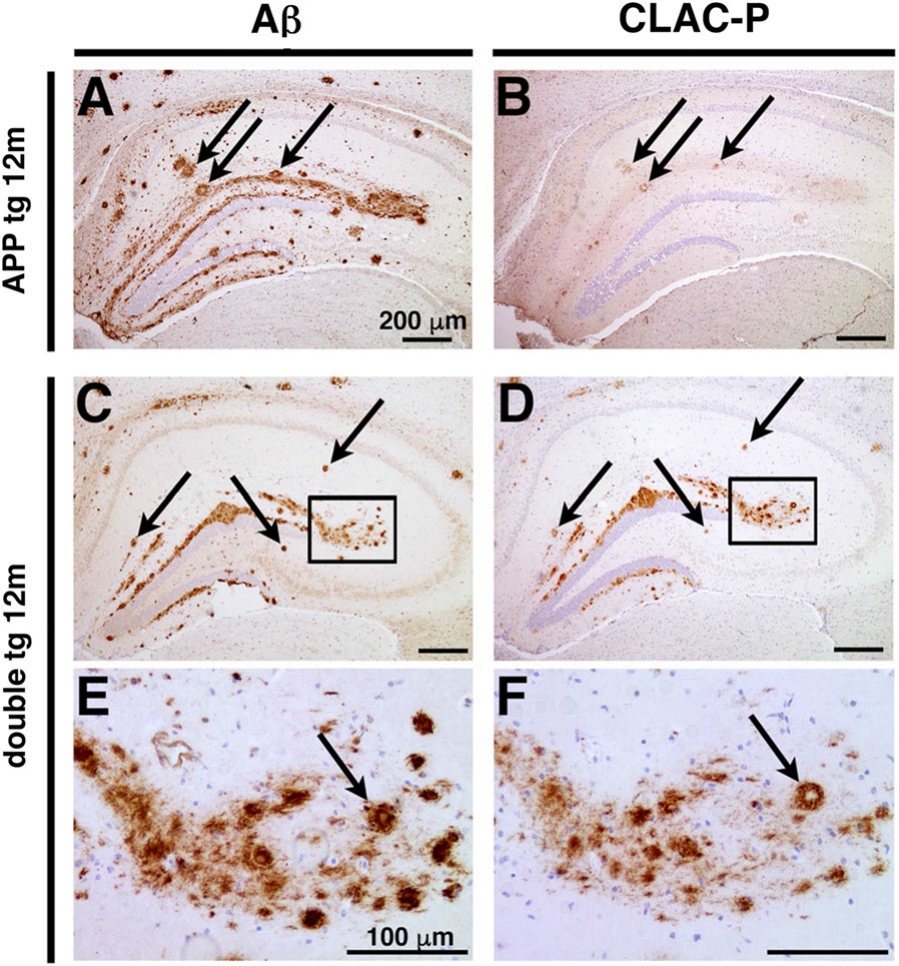
Endogenous and overproduced CLAC co-deposited with Aβ on the Aβ deposits. **a**–**f** Immunohistochemical analyses of the brains of 12-month-old APP tg (J20 line, **a** and **b**) and double tg mice (**c**–**f**) by anti-human Aβ (BAN50) (**a**, **c** and **e**) and anti-CLAC antibodies (anti-NC4) (**b**, **d** and **f**). High magnification images (insets in **c** and **d**) are shown in (**e**) and (**f**), respectively. In hippocampus of APP tg mice, immunoreactivities of murine endogenous CLAC were positive in a part of Aβ plaques (arrows in **a** and **b**). Middle-sized plaques in the hippocampus of double tg mice were visualized by anti-Aβ and anti-CLAC antibodies (arrows in **c**–**f**). Scale bar shows 200 μm (**a**–**d**) and 100 μm (**e** and **f**)

CLAC has been identified as an SP amyloid component in AD brains [18]. To examine whether overexpressed CLAC co-deposits with Aβ plaques in the brain, we immunostained serial sections from 12-months-old APP tg (J20 line) and their littermate double tg mice by anti-Aβ (BAN50) and anti-CLAC-P (anti-NC4) antibodies. We found that Aβ plaques in APP tg mice were weakly immunoreactive for endogenous CLAC (Fig. 4a, b), as previously observed in the brains of aged PS1/APP mice [27]. Numerous Aβ plaques in the hippocampus of double tg mice, especially most of the middle-sized compact plaques, were strongly positive for CLAC (Fig. 4c–f). Notably, within the core-positive plaques, the peripheral region was CLAC-positive, whereas the amyloid cores were devoid of CLAC immunoreactivities (Fig. 4e, f). Cerebrovascular amyloid deposits in the double tg mice also were CLAC-negative (data not shown). The patterns of distribution of CLAC in Aβ plaques were strikingly similar to those in AD brains [18, 27], supporting the notion that the interaction of CLAC with Aβ amyloid modifies the distribution and morphology of Aβ plaques in the brains of double tg mice, and possibly, senile plaques in the brains of patients with AD.

### Overexpression of CLAC-P promoted the maturation of Aβ plaques in the brains of double tg mice

**Fig. 5:**
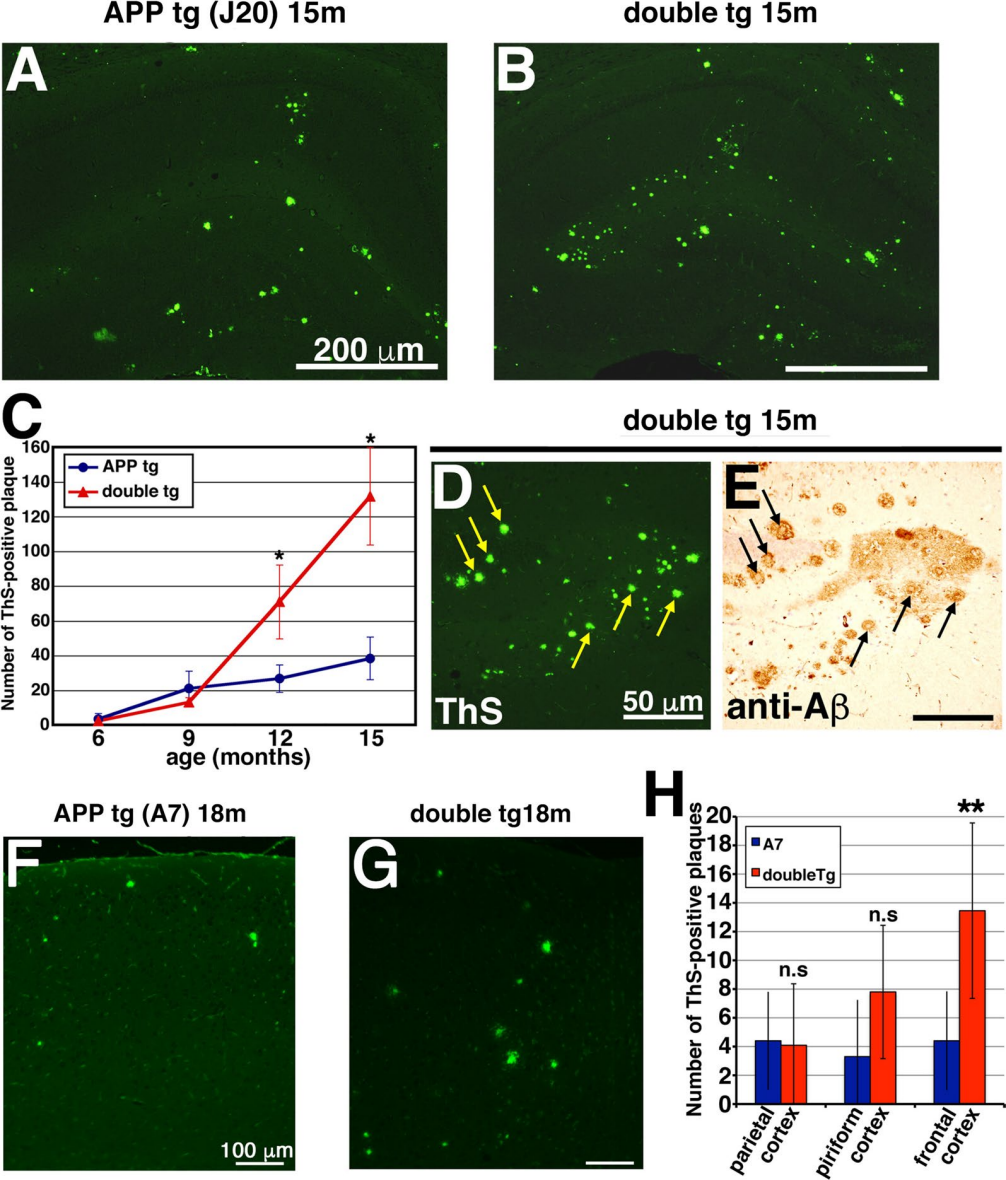
Overexpression of CLAC-P significantly increased the number of ThS-positive plaques. **a**, **b** ThS staining of the brains of 15-month-old APP tg (J20 line, **a**) and double tg mice (**b**). **c** The mean number of ThS-positive plaques was assessed in the hippocampus of 6-, 9-, 12- and 15-month-old APP tg mice (blue circles) and double tg mice (red triangles). N = 4, 4, 13, 7 for 6-, 9-, 12-, 15-month-old APP tg, respectively. N = 4, 3, 12, 7 for 6-, 9-, 12-, 15-month-old double tg, respectively. Student’s t-test, *p* = 0.81 (6-month-old), *p* = 0.24 (9-month-old), *p* < 0.0001 (12-month-old), *p* < 0.0001 (15-month-old). **d**, **e** Serial sections stained with ThS (**d**) and anti-Aβ antibody (BAN50) (**e**) were shown. The core-region of middle-sized plaques in the brains of 15-month-old double tg mice were exclusively labeled by ThS (arrows). **f**, **g** ThS staining of the brains of 18-month-old APP tg (**f**) and double tg (**g**) mice. **h** The mean number of ThS-positive plaques assessed in the parietal, piriform and frontal cortices of APP tg and double tg mice. N = 4, Student’s t-test, *p* = 0.86 (parietal cortex), 0.070 (piriform cortex), 0.00063 (frontal cortex), **, *p* < 0.01. Scale bar shows 200 μm (**a** and **b**), 50 μm (**d** and **e**), 100 μm (**f** and **g**)

To further examine the maturation of CLAC-positive Aβ plaques, we stained brains of 9 to 15-months-old APP tg (J20 line) and their littermate double tg mice with ThS (Fig. 5a, b), and found an age-dependent increase in the total numbers of ThS-positive Aβ plaques in the hippocampus of both APP single and double tg mice (Fig. 5c). Notably, numerous small-sized ThS-positive structures were visualized in the hippocampus of double tg mice at 15 months of age (Fig. 5d), which corresponded to the core region of compact plaques (Fig. 5e). The average number of ThS-positive plaques in the hippocampus was significantly higher in the double tg mice by ~ 2.65 times at 12 M (mean number per section, 26.7 in APP tg and 70.8 in double tg mice) and ~ 3.45 times at 15 M (38.2 in APP tg and 131.6 in double tg mice) compared with those in APP tg mice (Fig. 5c). We also examined the brains of 18-month-old APP tg (A7 line) and their littermate double tg mice with ThS and found that the average number of ThS-positive plaques in the piriform or frontal cortex of double tg mice also was significantly higher than those in APP tg mice (Fig. 5f–h). These data suggest that overexpression of CLAC-P promoted the compaction of Aβ plaques in the brains of double tg mice.

**Fig. 6:**
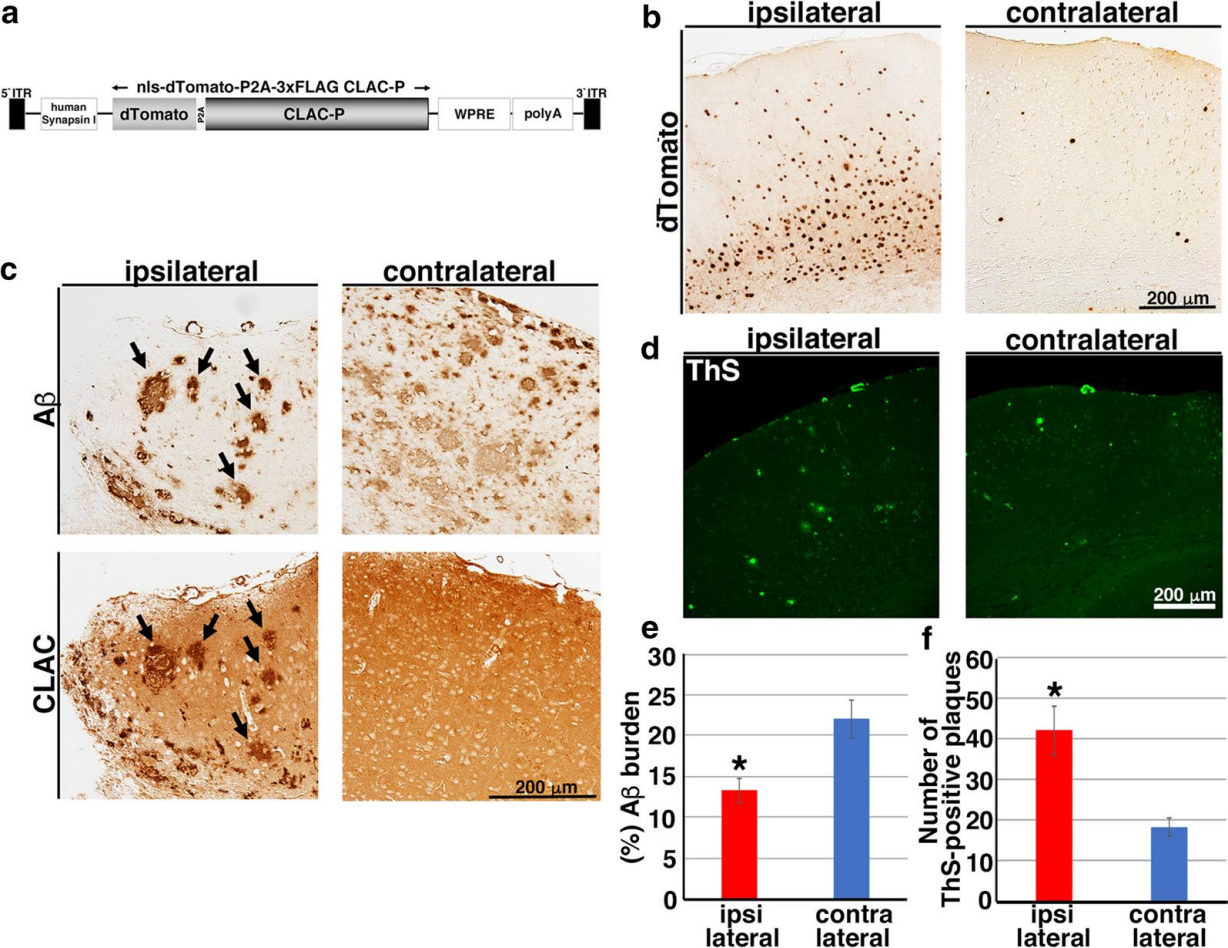
Overexpression of CLAC-P remodeled the morphology of pre-formed Aβ plaques. **a** A schematic structure of AAV9-CLAC-P. Under the human Synapsin I promoter, both dTomato and human CLAC-P are expressed bicistronically in neurons due to P2A self-cleaving peptide sequence. **b** Representative pictures of immunohistochemical staining of AAV9-CLAC-P-injected (ipsilateral) or PBS-injected (contralateral) hemisphere of 18-month-old APP tg mice (A7 line) using an anti-RFP antibody for dTomato. Scale bar shows 200 μm. **c** Immunohistochemical analyses of the brains of AAV9-CLAC-P-injected (ipsilateral) or PBS-injected (contralateral) 18-month-old APP tg mice (A7) using anti-Aβ (82E1, upper panels) or anti-CLAC antibodies (anti-NC3, lower panels). Arrows indicated the Aβ- and CLAC-double positive plaques. N = 5. Scale bar shows 200 μm. **d** ThS staining of the brains of AAV9-CLAC-P-injected (ipsilateral) or PBS-injected (contralateral) 18-month-old APP tg mice. Scale bar shows 200 μm. **e** Quantitative analysis of amyloid burden in the neocortex of AAV9-CLAC-P-injected (ipsilateral) or PBS-injected (contralateral) 18-month-old APP tg mice. N = 5, Paired t-test, *p* = 0.046, * *p* < 0.05. **f** The mean number of ThS-positive plaques in the neocortex of AAV9-CLAC-P-injected (ipsilateral) or PBS-injected (contralateral) 18-month-old APP tg mice. N = 5, Paired t-test, *p* = 0.017, * *p* < 0.05

To further examine whether CLAC remodels the morphology of pre-formed Aβ plaques in the brains of APP tg mice, we have adopted the AAV9 model to overexpress human CLAC-P in the neurons of plaque-bearing APP tg mice. We generated AAV9 vector carrying dTomato-P2A-CLAC-P (AAV9-CLAC-P, Fig. 6a) under the human Synapsin I promoter, which bicistronically expresses both dTomato and human CLAC-P in neurons. AAV9-CLAC-P or PBS was stereotaxically injected into the hippocampus and neocortex of 14-month-old APP tg mice (A7 line) that already harbor Aβ plaques both in the hippocampus and neocortex, to examine if expression of CLAC modifies the pre-existing Aβ plaques (Fig. 6b). Four months after injection, immunohistochemical analyses of the brains of APP tg mice showed dramatical changes of the morphology of Aβ plaques in the ipsilateral hemispheres injected with AAV9-CLAC-P: middle- or large- sized, well-circumscribed Aβ plaques positive both for Aβ and CLAC were seen, whereas amorphous diffuse-type Aβ plaques disappeared (Fig. 6c), similarly to the observation in the brains of double tg mice (Figs. 2, 3). Notably, a larger number of ThS-positive plaques were observed in the AAV9-CLAC-P-injected hemispheres compared with the PBS-injected side (Fig. 6d). Amyloid burden in the neocortices was significantly smaller in the neocortex ipsilateral to the injection side by ~ 40% (mean values: 13.3% in ipsilateral and 22.0% in contralateral neocortex) (Fig. 6e). The average number of ThS-positive plaques in the ipsilateral neocortex was significantly higher by ~ 2.3 times compared to that in the contralateral neocortex (41.9 in ipsilateral and 18.2 in contralateral neocortex) (Fig. 6f). These data supported the hypothesis that CLAC remodels the morphology of Aβ plaques into more compact and mature forms in the brain even after deposition.

**Fig. 7:**
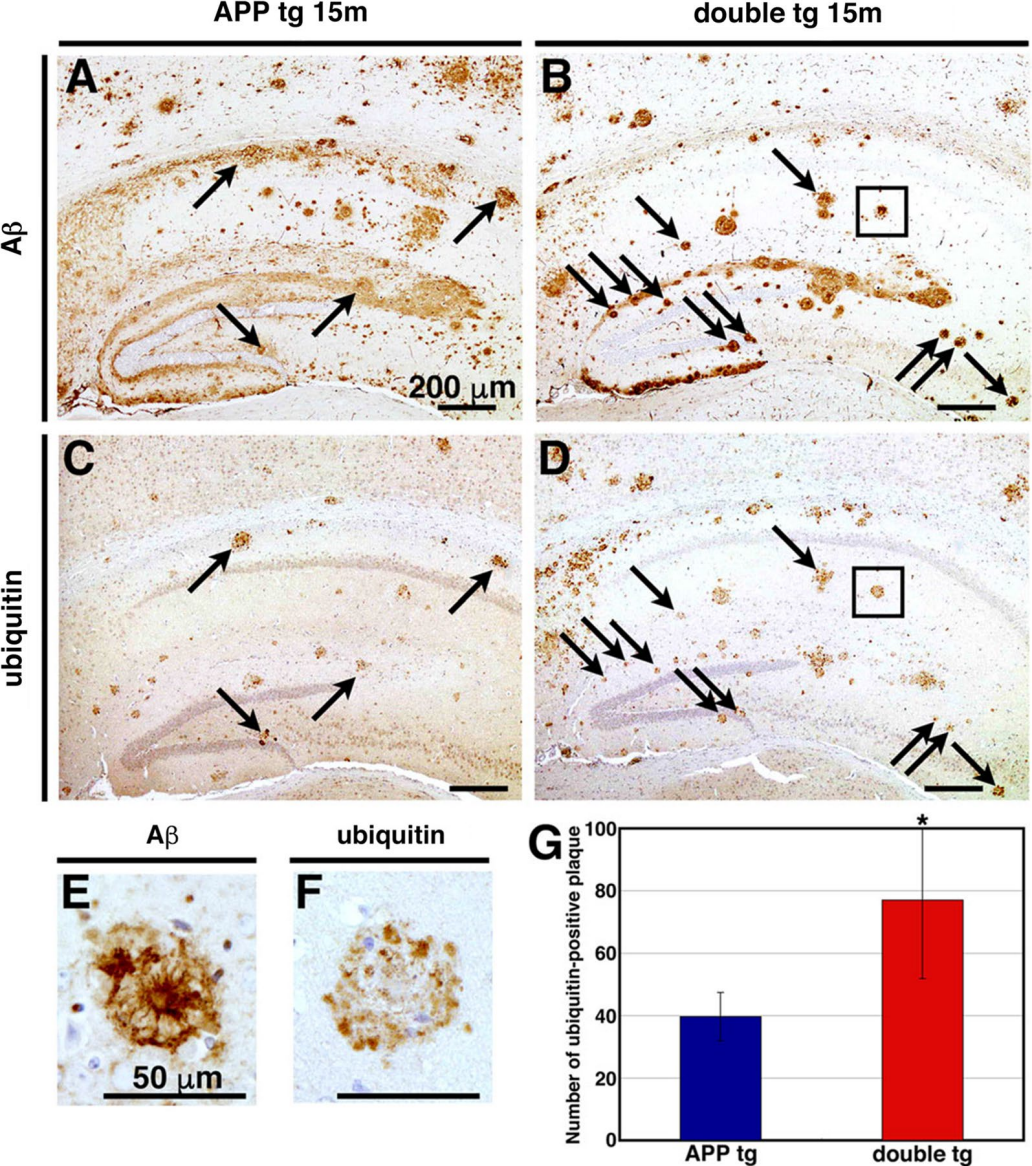
Overexpression of CLAC-P significantly increased the number of ubiquitin-positive neuritic plaques. **a**–**d** Immunohistochemical analyses of the brains of 15-month-old APP tg mice (J20 line, **a** and **c**) and double tg mice (**b** and **d**) using an anti-human Aβ antibody (BAN50) (**a** and **b**) and an anti-ubiquitin antibody (**c** and **d**). High magnification images (insets in **b** and **d**) were shown in **e** and **f**, respectively. A part of both Aβ and ubiquitin positive plaques were highlighted (arrows). Scale bar shows 200 μm (**a**–**d**) and 50 μm (**e** and **f**). **g** The mean number of ubiquitin-positive plaques was assessed in the hippocampus of 15-month-old APP tg mice (blue, N = 6) and double tg mice (red, N = 7). Mann–Whitney U test, *p* = 0.0066

Association of dystrophic neurites is a common feature of ThS-positive amyloid plaques in the brains of APP tg mice as well as of AD patients [9, 32]. To examine the association of dystrophic neurites with Aβ plaques in the brains of double tg mice, we immunostained serial sections from the brains of 15-month-old APP tg (J20 line) and their littermate double tg mice with an anti-Aβ (82E1) and anti-ubiquitin antibodies, the latter being a marker for dystrophic neurites, and found that numerous ubiquitin-positive, swollen neurites were present in the periphery of Aβ plaques in APP or double tg mice (Fig. 7). Notably, most of the middle-sized Aβ plaques in the double tg mice were decorated with ubiquitin immunoreactivities (Fig. 7b, d); typically, the periphery of the amyloid core-positive, middle-sized Aβ plaques were associated with numerous dystrophic neurites (Fig. 7e, f). The average number of ubiquitin-positive Aβ plaques in hippocampus of double tg mice was significantly larger by ~ 1.7 times compared with those in APP tg mice (40 Aβ deposits/section in APP tg and 77 in double tg mice, Fig. 7g).

Association of reactive microglial cells also is a characteristic feature of mature Aβ plaques [43]. To further examine the association of microglial cells in the brains of double tg mice, we immunolabelled serial sections from the brains of 15-month-old APP tg (J20 line) and their littermate double tg mice with an anti-Aβ (BAN50) and anti-Iba1 antibodies, the latter being a marker for microglial cells, and found the association of microglial cells at the periphery of Aβ plaques in APP or double tg mice (Additional file 1: Supplementary Fig. 1A). Notably, middle-sized, well-circumscribed Aβ plaques in the double tg mice were more densely associated with microglial cells than those in APP tg mice (Additional file 1: Supplementary Fig. 1A). The mean area of Iba1-immunoreactivities in hippocampus of 15 M double tg mice (J20 line) was larger by ~ 1.3 times compared with that in APP tg mice (4.06% in APP tg and 5.23% in double tg mice) (Additional file 1: Supplementary Fig. 1B), and that in the neocortex of 18 M double tg mice (A7 line) was significantly larger by ~ 6.8 times compared with that in APP tg mice (0.087% in APP tg and 0.59% in double tg mice) (Additional file 1: Supplementary Fig. 1C). These data suggest that overexpression of CLAC-P promoted the maturation of Aβ plaques, thereby increasing the plaque-associated local toxicity represented by the increase in dystrophic neurites or the association of microglial cells.

### Decreased level of Aβ in the brain interstitial fluid of double tg mice

**Fig. 8:**
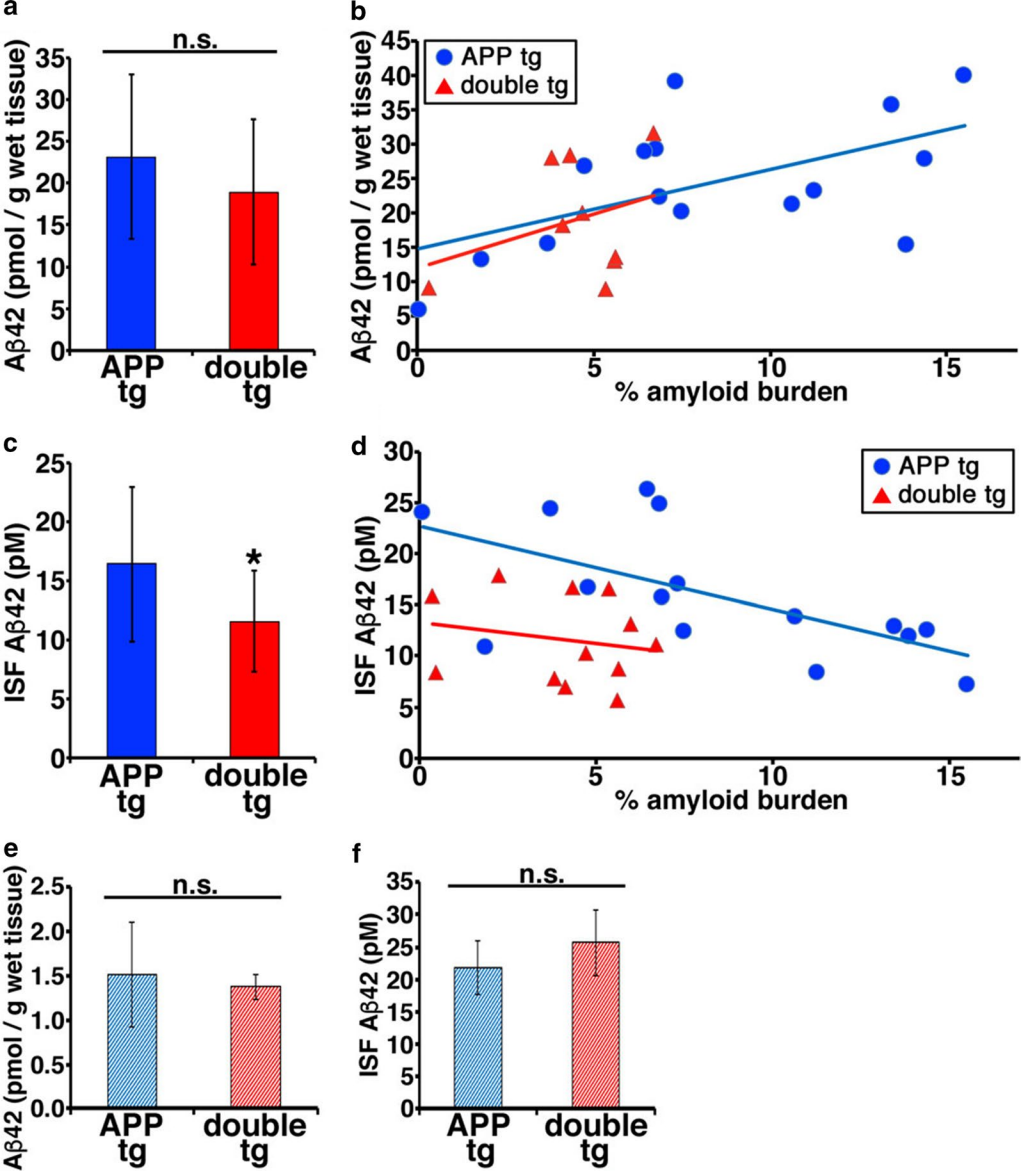
ISF Aβ in the brain of double tg was significantly decreased compared with APP tg. **a** The concentration of Aβ42 (pmol/g wet tissue) in the TBS-soluble fraction of the brains of 18–19-month-old APP tg (A7 line) and double tg mice. N = 13 (APP tg), and 9 (double tg). The mean ± SD Student’s t-test, *p* = 0.31 n.s. means no significant difference. **b** Correlation between the concentration of TBS-soluble Aβ42 and area of Aβ deposits in hippocamps of the brains of 18–22-month-old APP and double tg mice. R^2^ = 0.322 (APP tg), R^2^ = 0.108 (double tg). **c** The concentration of Aβ42 (pM) in brain ISF of 18–19-month-old APP and double tg mice. N = 13 (APP tg), and 12 (double tg). The mean ± SD Student’s t-test, *p* = 0.040. **d** Correlation between the concentration of ISF Aβ42 and area of Aβ deposits in hippocamps of the brains of 18–22-month-old APP and double tg mice. R^2^ = 0.385 (APP tg), R^2^ = 0.0416 (double tg). **e** The concentration of Aβ42 (pmol/g wet tissue) in the TBS-soluble fraction of the brains of 5–7-month-old APP and double tg mice. N = 4 (APP tg), and 4 (double tg). The mean ± SD Welch’s t-test, *p* = 0.67. n.s. means no significant difference. **f** The concentration of ISF Aβ42 (pM) in the brains of 5–7-month-old APP and double tg mice. N = 4 (APP tg), and 4 (double tg). The mean ± SD Student’s t-test, *p* = 0.28. n.s. means no significant difference

We next sought to examine whether the maturation of Aβ plaques induced by association of CLAC affected the dynamics of soluble Aβ in the brain parenchyma. To this end, we quantified the levels of Aβ42 in the brain interstitial fluid (ISF) by in vivo microdialysis technique using a 1,000 kDa molecular weight cut-off microprobe [7, 59], which represents soluble, diffusible, < 1000 kDa Aβ species present in the brain extracellular space. We also quantitated the levels of Aβ42 extracted in TBS that corresponds to total soluble Aβ in the brain. The levels of TBS-soluble Aβ42 in the brain were similar between the 18–22-month-old APP tg (A7 line) and its littermate double tg mice (23.1 pM in APP tg and 18.9 pM in double tg mice, Fig. 8a). On the scatterplot analysis, we found a positive correlation between the levels of TBS-soluble Aβ42 and the percentage area of Aβ burden in the APP tg mice, but not in the double tg mice (R^2^ = 0.322 in APP tg and R^2^ = 0.108 in double tg mice, Fig. 8b). In sharp contrast, we found that the levels of ISF Aβ42 in the hippocampus of 18–22-month-old double tg mice were significantly lower than that of APP tg mice (16.5 pM in APP tg and 11.6 pM in double tg mice, Fig. 8c). We also found a negative correlation between ISF Aβ42 and the percentage area of Aβ burden in the APP tg mice, but not in the double tg mice (R^2^ = 0.385 in APP tg and R^2^ = 0.042 in double tg mice, Fig. 8d). These results suggest that ISF Aβ may be captured by Aβ deposits and sequestered from ISF, and consequently, CLAC-positive, more compact Aβ plaques in the brains of double tg mice might have sequestered soluble Aβ from the ISF to a greater extent compared with that in APP tg mice. To verify this idea, we quantified the levels of TBS-soluble brain Aβ42 and ISF Aβ42 in 5–7-month-old APP tg mice and its littermate double tg mice that have not developed plaques yet. Comparable levels of TBS-soluble brain Aβ42 and ISF Aβ42 in the hippocampus were detected between APP tg and double tg mice (Fig. 8e, f). Taken together, we concluded that CLAC might be one of the determinants of the morphology of Aβ plaques, which promotes the compaction of Aβ deposits, thereby altering the dynamics of soluble Aβ in the brain.

### CLAC impacts the compaction of Aβ plaques without influencing the total amount of insoluble Aβ

**Fig. 9:**
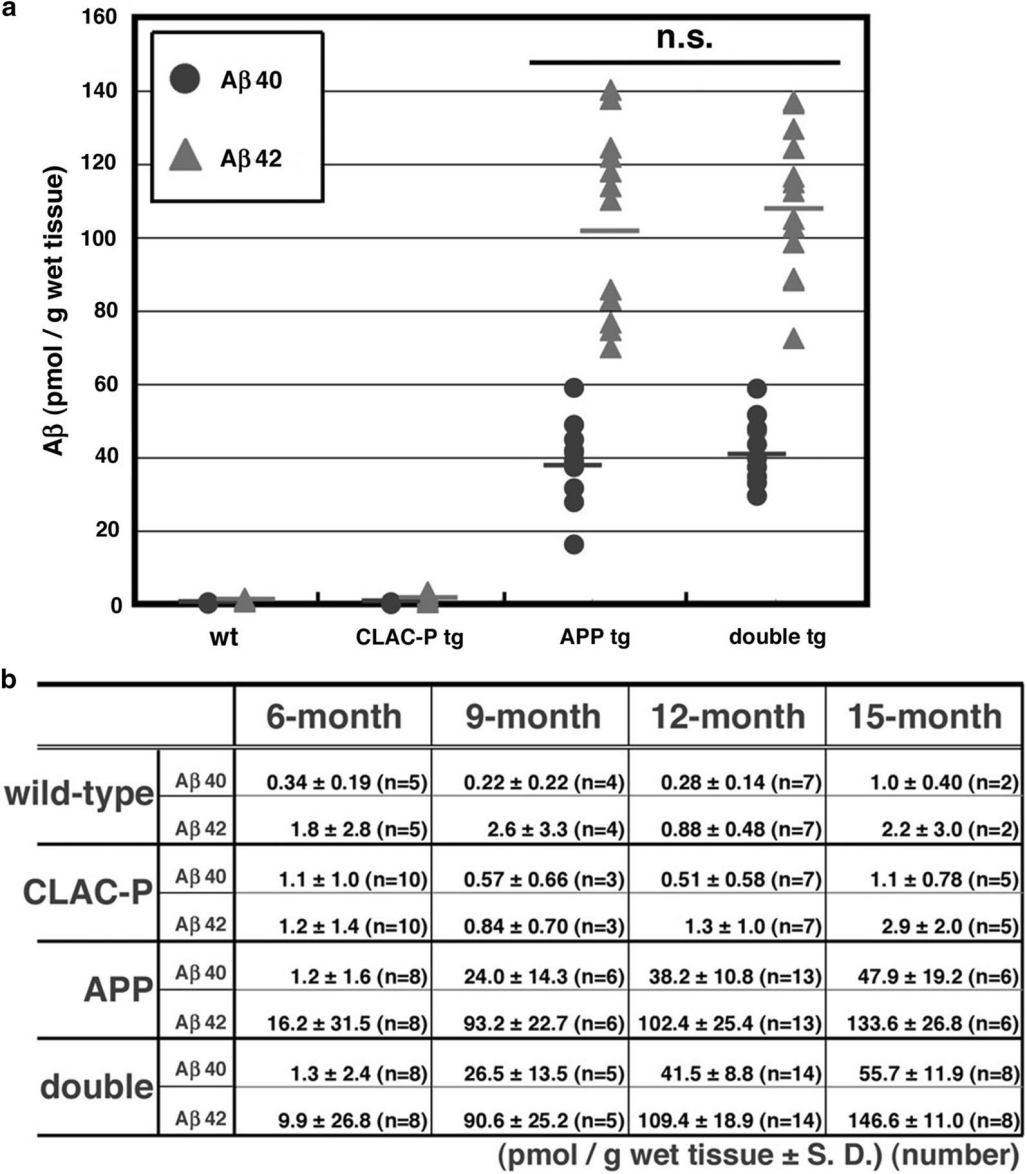
Similar levels of insoluble Aβ in the brains of APP and double tg mice. **a** The concentration of Aβ40 (circles) and Aβ42 (triangles) in the 2% SDS-insoluble, 70% formic acid-soluble fraction of the brains of 12-month-old wild-type (N = 7), CLAC-P tg (N = 7), APP tg (J20 line, N = 13) and double tg (N = 14) mice were measured by a two-site ELISA. Student’s t-test, *p* = 0.40 (Aβ40 in APP tg vs Aβ40 in double tg), 0.44 (Aβ42 in APP tg vs Aβ42 in double tg). n.s. means no significant difference. **b** The levels of Aβ40 and Aβ42 in the SDS-insoluble, 70% formic acid-soluble fraction of the brains of 6-, 9-, 12- and 15-month-old double tg mice and littermates. The mean ± SD of the amount of Aβ40 and Aβ42 are shown

To ask whether CLAC influenced the levels of insoluble Aβ in vivo, we quantified Aβ40 and Aβ42 in the formic acid extracts of the SDS-insoluble fraction from the brains of 6, 9, 12 or 15-month-old wild type, CLAC-P tg, APP tg (J20 line) or littermate double tg mice by ELISAs. The levels of SDS-insoluble, formic-acid soluble human Aβ40 and Aβ42 in the brains of APP tg mice started to increase at ages of 9 and 6 months, respectively (Fig. 9). No significant differences in the levels of SDS-insoluble, formic-acid soluble Aβ40 or Aβ42 in the brains were found between APP and double tg mice. We also found that the levels of SDS-insoluble, formic-acid soluble Aβ40 and Aβ42 in the brains of 18-month-old APP tg (A7 line) and littermate double tg mice (data not shown) also were similar. These data suggested that CLAC did not affect the total amount of insoluble Aβ in the brain.

Lastly, we asked whether co-deposition of CLAC on the Aβ plaques influences the pattern of distribution in Aβ deposition. A number of CLAC-positive small deposits were observed in the neocortices of CLAC-P tg (Fig. 1e) or double tg (J20 line, Additional file 1: Supplementary Fig. 2B, D) mice. Ultrastructural analysis showed that extracellular filamentous aggregates positively labeled by an anti-CLAC-P antibody (NC4) were present in the cortex of double tg mouse (Additional file 1: Supplementary Fig. 2E). We confirmed that these CLAC-positive deposits were entirely Aβ-negative (Additional file 1: Supplementary Fig. 2). This may suggest that CLAC aggregates do not directly interact with secreted non-fibrillar Aβ and elicit deposition of Aβ fibrils.

## Discussion

To examine the roles of CLAC as an Aβ-plaque associated protein in the plaque formation and Aβ dynamics in brains, we have generated tg mice that doubly overexpress human CLAC-P and APP, and observed a dramatic increase in compact plaques with ThS-positive cores, a decrease in diffuse plaques, and a significant decrease in the ISF Aβ levels in the double tg compared with the APP single tg mice. In contrast, the levels of SDS-insoluble, formic acid-soluble Aβ, as well as those of TBS-soluble Aβ, were comparable between the single and double tg mice. Based on these data, we speculated that CLAC impacted on the process of amyloid plaque formation, specifically by promoting the compaction of Aβ deposits, leading to the maturation of Aβ plaques as well as an alteration in the dynamics of Aβ in the interstitial fluids.

Although we found that the disappearance of diffuse type Aβ plaques and an increase in the number of compact Aβ plaques with ThS-positive amyloid cores in the brains of double tg mice and AAV9-CLAC-P-infected APP tg mice, it is unclear how CLAC alters the morphology of Aβ plaques in the brain. One possible mechanism would be that CLAC pre-existed within plaques further attracted Aβ fibrils onto the plaques, resulting in an increase in the density of Aβ fibrils and compaction of amyloid plaques. Our previous findings that CLAC specifically interacted with fibrillized form of Aβ [18, 37], and that CLAC was exclusively positive on amyloid bundles in the primitive or typical SPs in AD brains, but not on the diffuse type SPs [27], may support this view. However, we have observed a number of CLAC-positive small deposits within the entire neocortices of CLAC-P tg or double tg mice that were entirely negative for Aβ; the finding that CLAC aggregates per se do not directly elicit Aβ deposition in the brain parenchyma may imply that CLAC/Aβ co-deposits that harbor a specific conformation that attracts Aβ fibrils might be involved in the amyloid compaction.

Another possible scenario would be that CLAC deposited with Aβ in the periphery of plaques drives the deposition of Aβ fibrils concentrically into amyloid plaques, leading them to closely pack at the center of amyloid plaques. In this context, it is noteworthy that the binding of CLAC has been suggested to confer protease resistance to amyloid fibrils [44]. It is tempting to speculate that CLAC associated with Aβ plaques affects the microglial phagocytosis or proteolysis of amyloid fibrils, resulting in the remodeling and compaction of Aβ plaques.

We have shown that overexpression of CLAC-P impacts on the compaction of Aβ plaques in the brain, although the levels of CLAC polypeptides in the brain required for the compaction of Aβ plaques remained to be estimated. To address this question, further biochemical and histochemical studies using APP tg mice injected with various concentrations of AAV9-CLAC-P may be required. It may also be critical to investigate whether the deficiency in murine *CLAC*-*P* gene decreases the maturation of Aβ plaques in the brains of APP tg mice. We have previously reported that CLAC-P deficient mice exhibited perinatal lethality due to the incomplete formation of neuromuscular junctions [50]. Cross of APP tg mice with adult neuron-specific conditional *CLAC*-*P* knockout mice will provide us with an answer to the question.

It also remains an open question whether the co-deposition of CLAC on SPs together with Aβ acts deteriorative or protective in the pathophysiology of AD. We found a significant increase in the number of ThS-positive plaques, ubiquitin-positive dystrophic neurites and microglial cells in the brains of double tg mice compared with the APP single tg. It has been speculated that ThS-positive plaques are associated with local neurotoxicity [52] or affect the neurite trajectories and synaptic structures compared with ThS-negative Aβ plaques in the brains of patients with AD and APP tg mice [5, 9, 32]. These results prompted us to speculate if the brains of double tg mice may suffer from higher vulnerability to neurodegeneration compared with APP tg mice. Altogether, CLAC may play an important role in the remodeling of Aβ plaques into matured plaques in relation to the pathogenesis of AD. Further behavioral studies and more quantitative analysis of neurons and synapses in tg brains may provide us with further clues to the significance of CLAC in amyloid plaques.

In the brain of APP tg mice, we found a positive correlation between the levels of TBS-soluble Aβ42 and the area of Aβ burden, whereas the levels of ISF Aβ42 and the areas of Aβ burden were negatively correlated. These apparently contradictory results led us to hypothesize that SPs might have dual functions against soluble Aβ as a sink and a reservoir in the brain: SPs may sequester soluble Aβ from ISF and retain Aβ on the surface of the plaques. Thus, the levels of ISF Aβ are decreased and those of TBS-soluble Aβ increased, as Aβ accumulates on plaques in the brain. In accordance with the classical observation that soluble radiolabeled Aβ(1–40) is accumulated on SPs in frozen tissue sections from AD brains in vitro [30], it has been shown that exogenous administration of soluble radiolabeled Aβ(1–40) into the brains significantly lowers the recovery rate of Aβ in the ISF of plaque-rich APP tg mice compared with that in plaque-free mice [21]. In vivo stable isotope-labeling kinetics studies in AD patients showed that the presence of amyloid plaques led to a dramatic slowing of Aβ turnover [38, 39]. Taken together with our present data, one could argue that the amyloid plaques act as a reversible exchange pool of soluble Aβ in the brain, which impacts on the dynamics of soluble Aβ. We have observed a significantly lower ISF level of Aβ in the brains of plaque-rich double tg mice compared with APP single tg mice, although the levels of Aβ recovered in the TBS-soluble fraction were similar. In contrast, the levels of ISF Aβ in the brains of plaque-free double tg mice was comparable to those in APP tg mice. These data suggest that the maturation of Aβ plaques, which was caused by overexpression of CLAC-P in the double tg mice in the present study, might have promoted the sequestration of soluble Aβ from ISF onto the Aβ plaques. This also supports our speculation that the morphology of amyloid plaques, along with the amount of amyloid deposition, is a determinant of the dynamics of soluble Aβ in the brain extracellular milieu.

A number of non-Aβ protein components have been identified in SPs other than CLAC [11]. Apolipoprotein E (apoE), whose polymorphism has been identified as a major genetic risk factor for AD [8, 40, 46], is co-deposited with Aβ in AD brains [36]. Ablation of *APOE* gene in the APP tg mice caused a dramatic decrease in the number of Congo red or ThS-positive fibrillar Aβ deposits [3, 4, 49], and an increase in the diffuse-type Aβ deposits [19, 20, 53]. It is noteworthy that the levels of ISF Aβ as well as cerebrospinal fluid Aβ in the brains of plaque-free *APOE*–/– APP tg mice was significantly increased compared with those of APP tg mice [10]. It seems likely that apoE influences the dynamics of soluble Aβ and facilitates the conversion of soluble Aβ into forms with a high β-sheet content. We found no changes in the level of ISF Aβ in the brains of plaque-free APP/CLAC-P double tg mice compared with the APP single tg mice. This suggests that CLAC affects the morphology of Aβ plaques and the brain dynamics of soluble Aβ in a different manner from that of apoE.

In sum, we have shown that the association of CLAC contributes to the maturation of Aβ plaques in the brain, which may mediate the local toxicity around the deposits and impact the dynamics of soluble Aβ, using a mouse model of AD overexpressing CLAC-P and APP. These findings will give us clues as to the origin of different forms of Aβ plaques in AD brains, and further provide a novel therapeutic target for AD through suppression of SP maturation, for example, by inhibiting the interaction between CLAC and Aβ fibrils.

## Conclusion

We reveal that CLAC remodels Aβ plaques into more compact and mature forms, and reduces the soluble Aβ species in the interstitial fluid in the brains of APP transgenic mice. CLAC acts as a molecular determinant of the Aβ plaque morphology, which consequently alters the dynamics of Aβ presumably by sequestering the soluble Aβ from brain extracellular space to plaques.

## Supplementary information

**Additional file 1.** Supplementary methods. Supplementary **Fig. 1**: Immunohistochemical analyses using an antibody to microglia; Supplementary **Fig. 2**: Immunnohistochemical analyses and ultrastructure of CLAC-positive cortical deposits.

## Data Availability

The datasets and materials used and/or analyzed during the current study are available from the corresponding author on reasonable request.

## Abbreviations

AAV9: Adeno-associated virus serotype 9
aCSF: Artificial cerebrospinal fluid
AD: Alzheimer’s disease
Aβ: Amyloid-β peptide
APP: Aβ precursor protein
CLAC: Collagenous Alzheimer amyloid plaque component
CLAC-P: CLAC precursor protein
COL25A1: Collagen type XXV α1 chain
DMSO: Dimethyl sulfoxide
ELISA: Enzyme-linked immunosorbent assay
ISF: Interstitial fluid
PBS: Phosphate-buffered saline
SP: Senile plaque
TBS: Tris-buffered saline
tg: Transgenic
ThS: Thioflavin S
