# Supplementary material for: Collagenous Alzheimer amyloid plaque component impacts on the compaction of amyloid-β plaques
Source: Acta Neuropathol Commun. 2020 Dec 7;8:212. doi: 10.1186/s40478-020-01075-5 (PMC7720522; doi:10.1186/s40478-020-01075-5)

Collagenous Alzheimer amyloid plaque component impacts on the compaction of amyloid-β plaques

Tadafumi Hashimoto, Daisuke Fujii, Yasushi Naka, Mayu Kashiwagi-Hakozaki, Yuko Matsuo, Yusuke Matsuura, Tomoko Wakabayashi and Takeshi Iwatsubo

Supplementary method

Immunoelectron microscopy

Immunogold labeling for electron microscopic observation of CLAC in double tg mice was performed as described previously (Kowa et al, 2004). Briefly, 50-μm thick, floating sections fixed in 50 mM phosphate buffer containing 4% paraformaldehyde (pH 7.4) for 24 hrs were incubated with an anti-CLAC-P antibody (NC4) overnight. After washing, sections were incubated with anti-rabbit IgG antibody tagged with 1-nm gold particles (Nanoprobe) for 24 hrs. After washing, sections were post-fixed in 3.5% glutaraldehyde (EM Science) for 1 hr, and transferred to 2-[4-[2(hydroxyethyl)-1-piperazinyl] ethanesulfonic acid buffer (pH 5.8). After washing in distilled water, silver intensification was performed using HQ-silver kit (Nanoprobes) according to manufacturer's instructions. After stopping the silver intensification by washing in distilled water, the sections were post-fixed in 2% osmium tetroxide for 1 hr, dehydrated, and embedded in epoxy resin. Ultra-thin sections were cut at 80 nm, double-stained by uranium-acetate and lead-citrate, and viewed in electron microscope (1200EXII, JEOL).

Supplementary Figure legends

Supplementary Figure 1. Overexpression of CLAC significantly increased the association of microglial cells with Aβ plaques

(A) Immunohistochemical analyses of the brains of 15-month-old APP tg mice (J20 line, left row) and littermate double tg mice (right row) with an anti-human Aβ antibody (BAN50) or an anti-Iba1 antibody on serial sections. Middle-sized, well-circumscribed Aβ plaques associated with microglial cells are highlighted by red arrows (right row). Scale bar shows 50 μm. (B, C) Quantitative analysis of Iba1-positive area in the hippocampus of 15-month-old APP tg and double tg mice (J20 line, B), or in the neocortex of 18-month-old APP tg and double tg mice (A7 line, C). (B) N= 6 (APP), and 7 (double),

Student's t-test, $p=0.37$. (C) N=7 (APP), and 7 (double), Student's t-test, $p=0.038$, * $p<0.05$.

Supplementary Figure 2. CLAC- positive cortical deposits are entirely negative for Aβ in the brains of double tg mice

(A-D) Immunohistochemical analyses of the brains of 12-month-old double tg (J20 x CLAC-P) mice using an anti-human Aβ antibody (82E1) (A and C) and an anti-CLAC-P antibody (anti-NC4) (B and D). High magnification images of the boxed area in A and B are shown in (C) and (D), respectively. A subset of Aβ-positive plaques (arrows in C) were positively labeled with anti-CLAC-P antibodies (arrows in D). In contrast, small plaque-like structures immunolabeled by anti-CLAC-P antibody in the neocortex (arrowheads in D) were entirely negative for Aβ (arrowheads, C and D). Scale bar shows 200 μm. (E) Ultrastructure of the CLAC-positive deposits in the cortex of double tg mice. Bundles of extracellular filamentous structures are labeled with an anti-CLAC-P antibody (NC4), as visualized by 1 nm immunogold/silver intensification. Scale bar = 500 nm.

Supplementary Figure 1

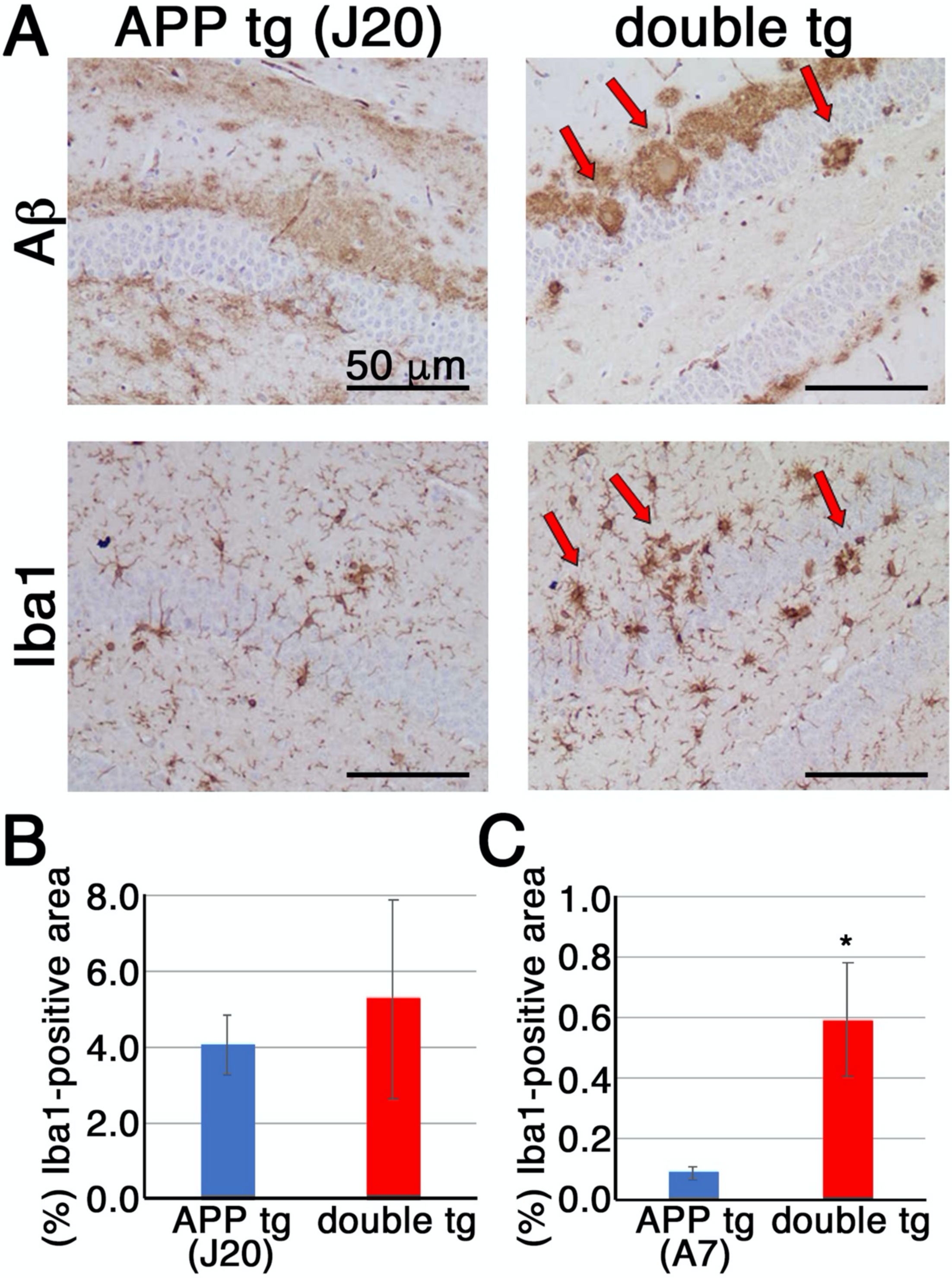

Supplementary Figure 2

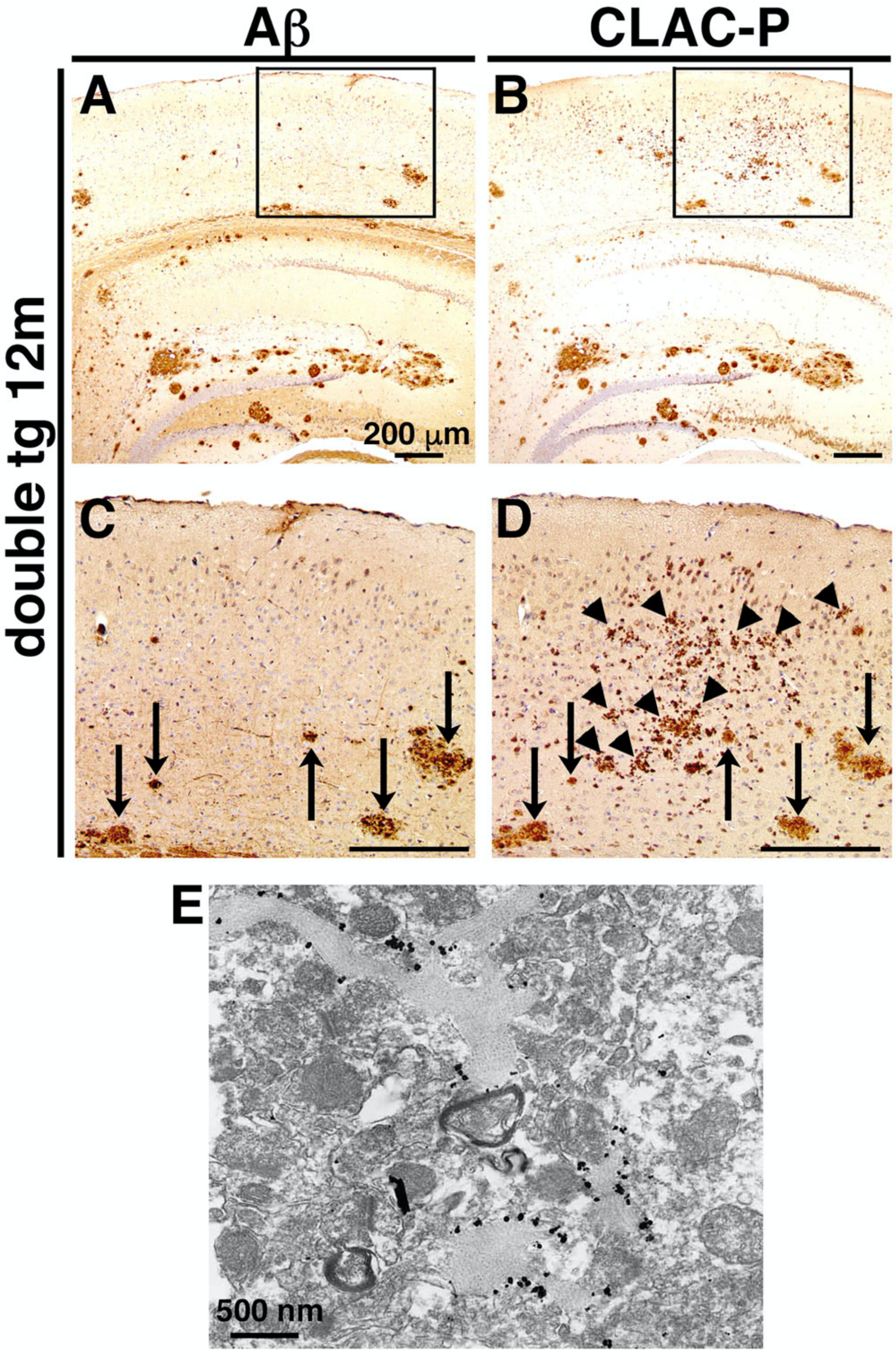

Supplement: Supplementary file 1 — Additional file 1. Supplementary methods. Supplementary Fig. 1: Immunohistochemical analyses using an antibody to microglia; Supplementary Fig. 2: Immunnohistochemical analyses and ultrastructure of CLAC-positive cortical deposits. [file 40478_2020_1075_MOESM1_ESM.pdf]
